# Lattice Dynamics of the Rhenium and Technetium Dichalcogenides

**DOI:** 10.1186/s11671-016-1459-9

**Published:** 2016-05-13

**Authors:** Daniel Wolverson, Lewis S. Hart

**Affiliations:** Department of Physics, University of Bath, Claverton Down, Bath, UK

**Keywords:** Rhenium sulphide, Technetium sulphide, Rhenium selenide, Transition metal dichalcogenide, Raman spectroscopy

## Abstract

The rhenium and technetium dichalcogenides are layered van der Waals semiconductors which show a large number of Raman-active zone-centre phonon modes as a result of their unusually large unit cells and deviation from hexagonal symmetry. They thus offer the possibility of introducing in-plane anisotropy into composite heterostructures based on van der Waals materials, and Raman spectroscopy is generally used to determine their in-plane orientation. We show that first-principles calculations give a good description of the lattice dynamics of this family of materials and thus predict the zone-centre phonon frequencies and Raman activities of TcS_2_. We consider the distribution of the phonon modes in frequency and their atomic displacements and give a unified understanding of the phonon frequencies and Raman spectra of ReS_2_, TcS_2_ and ReSe_2_ in terms of the scaling of Raman frequency with the chalcogen mass.

## Background

The transition metal dichalcogenides (TMD) have been known for a considerable time; the basic properties of about 40 members of the family were reviewed in 1969 [1]. However, the enormous fundamental scientific interest generated by the isolation of graphene, and the potentially disruptive technologies based on it, has stimulated interest in the wider family of two-dimensional layered van der Waals materials, to which the TMDs belong, with some 2500 publications on the archetypal family member MoS_2_ alone in 2015. This level of activity is likely to be sustained as researchers develop the concept of devices based on multi-layered structures [2] in which dissimilar materials are stacked to achieve new functionalities, for instance, as light emitters [3, 4]. The key to this approach is the wide variety of behaviors that the TMDs exhibit, with metallic, semiconducting and superconducting phases, with properties that can be tuned by doping, and with novel types of coupling between spin and valley physics [5]. Here, we consider some of the less well-studied TMDs, based on Re and Tc, which are also attracting growing interest.

The rhenium and technetium dichalcogenides are layered van der Waals semiconductors with large unit cells due to their deviation from hexagonal symmetry towards a distorted 1T ^′^ structure with space group P$\bar {1}$ in which the metal atoms group into parallelograms of four atoms [6–8]. They thus offer the unusual possibility of introducing a built-in planar anisotropy into composite heterostructures. Clearly, if their anisotropy is to be useful, one must be able to determine their in-plane orientation, and one promising method for this is Raman spectroscopy [9] since the large unit cell and lack of symmetry-related degeneracies leads to a large number (18) of Raman-active zone-centre phonon modes [9, 10].

It has already been shown that first-principles calculations can give a good description of the lattice dynamics of this family of materials [9, 10]; here, we compare predictions of the phonon frequencies of ReS_2_, TcS_2_ and ReSe_2_. The zone-centre phonon modes show a common distribution in frequency, and we give a simple interpretation of this in terms of the atomic displacements, which leads to a unified understanding of the Raman spectra of ReS_2_, TcS_2_ and ReSe_2_.

Little is known directly about crystalline TcS_2_ though its structure is known [6, 8] and its electronic band structure has been predicted via density functional theory [11–13]. Technetium is not found naturally on Earth but only occurs as a by-product of nuclear fission of uranium-235; all its isotopes are unstable. The most readily available isotope, ^99*m*^Tc, is used in nuclear medicine as a radiopharmaceutical for labeling applications and is highly radioactive with a half-life of ∼6 h. In that context, it is sometimes produced in the form of a colloid with rhenium sulphide [14]. A longer-lived isotope is ^99^Tc (half-life 2.13 × 10^5^ years), and the planning of long-term control and storage for this radionuclide in a water-insoluble form is a priority. One proposed strategy involves sequestration of ^99^Tc by the formation of its sulphides [15]. For both these applications, a knowledge of the Raman spectrum of TcS_2_ should provide a useful analytic and diagnostic tool. We therefore also present a prediction of the Raman-active modes of TcS_2_ and show how these relate to those of the analogous rhenium compounds.

## Methods

**Fig. 1:**
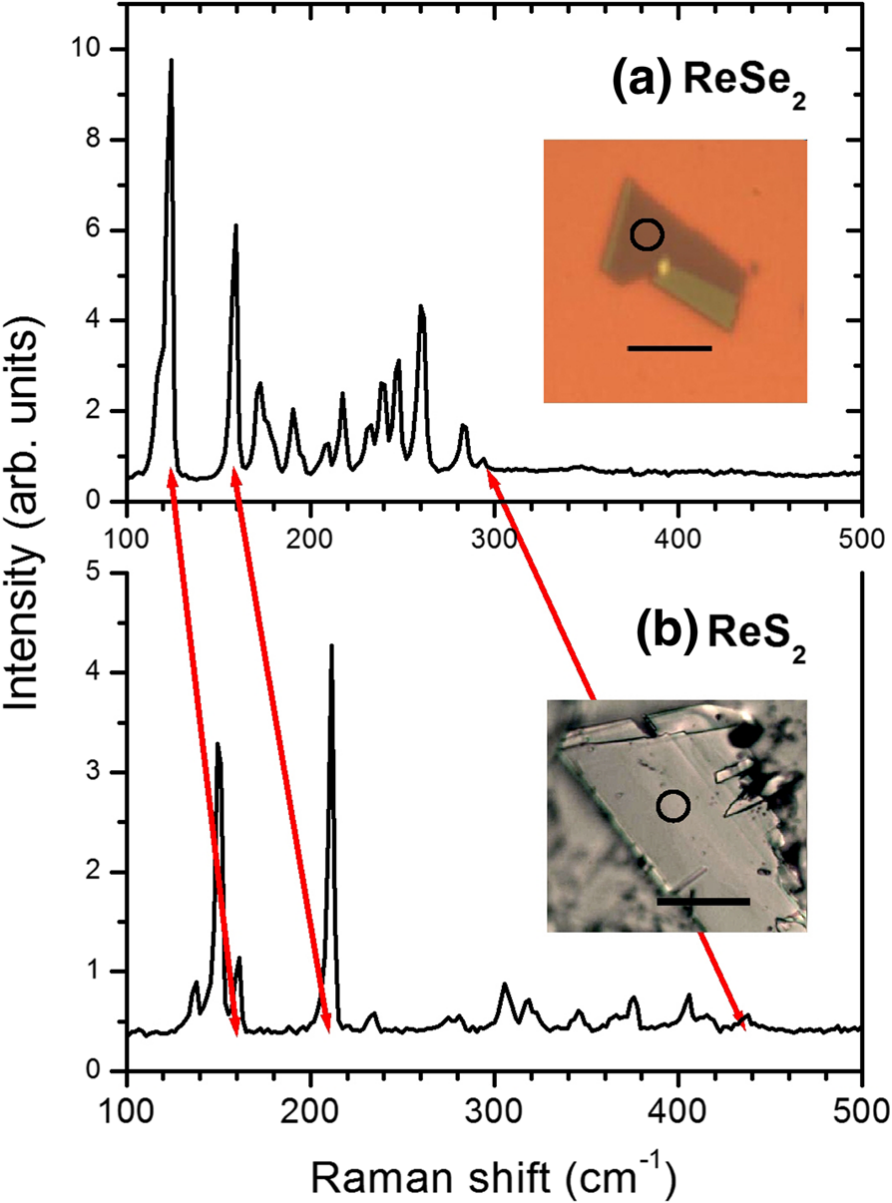
The Raman spectra of thick **a** ReSe_2_ and **b** ReS_2_ flakes on a SiO_2_/silicon substrate at room temperature; the *red arrows* link the peaks in the two materials corresponding to three phonon modes of significance for the present discussion. The *insets* show the the sample flakes used; the *circles* show the areas sampled and the *bars* indicate a scale of 20 *μ*m

Raman spectra in a backscattering geometry were obtained for the rhenium dichalcogenides using a Renishaw InVia Raman microscope with a ×50 objective lens giving a laser spot size of around 1 *μ*m with, typically, 100 *μ*W of 532-nm excitation; this microscope also provided the images of the samples in Fig. 1. The preparation (by micro-mechanical cleavage) and characterization of the ReSe_2_ samples is described elsewhere [9], and the ReS_2_ sample was prepared similarly. The ReSe_2_ sample was a few unit cells in thickness whereas the ReS_2_ sample was thicker; the differences between monolayer, few-layer and bulk Raman spectra of these materials are small [9, 10], being much less significant than in the case of MoS_2_, for example, but have recently been shown to vary systematically with thickness, as do the interlayer modes [16–19].

Calculations of phonon frequencies were carried out using density functional perturbation theory [20] (DFPT) as implemented in the Quantum Espresso code [21]. Initial atomic coordinates were taken from earlier X-ray diffraction studies [7, 8] and were relaxed to reduce the atomic forces below 10^−3^ eV/Å. We have explored the use of norm-conserving pseudopotentials (NCPP) and also the projector augmented wave (PAW) method within both the local density (LDA) and generalized gradient (GGA) approximations; the resulting phonon frequencies given by these methods vary by less than ±2 % and are in good agreement with experiment, as found by other authors [10, 22]. The criteria for the convergence of the total energy with respect to kinetic energy cutoffs (here typically 680 eV) and *k*-point grid size (typically 6×6×6 for 3D or 6×6×1 for 2D) were discussed earlier [9]. Since the bulk unit cell of ReS_2_ may be doubled along the out-of-plane axis [8] (that is, the unit cell may contain two layers), we calculated the phonon modes for an isolated monolayer with a single layer per supercell, so as to obtain the same number of modes and thus facilitate comparison. Since the differences between the phonon modes of monolayers and multiple layers are small for a transition metal dichalcogenide [10, 23], this procedure is justified. We note also that this question is not resolved, and one group saw no evidence of doubling of the unit cell for ReS_2_ [24].

## Results and Discussion

Figure 1 shows representative spectra of ReSe_2_ and ReS_2_; most of the 18 expected Raman-active *A*_*g*_ modes can be seen and, as has been described in detail before, the peak intensities are a function of the angle of orientation between the excitation laser polarization and the in-plane crystal axes [9, 18]. For these low-symmetry materials, all components of the Raman tensors for each *A*_*g*_ mode are non-zero, so that all Raman-active modes have a finite intensity at any angle, but the maximum intensities of different modes occur at different angles. The modes are densely spaced in frequency, but for both materials there is a noticeable “gap” in the spectrum between the 9th and 10th modes as indicated by the red arrows in Fig. 1 (the higher red arrow links the positions of the highest-frequency Raman-active modes). This makes the two modes in either side of this gap easy to recognize even when weak; they are also found to be amongst the strongest modes in the Raman spectrum [25–28], and so have been a focus of other studies, where they are sometimes labelled as *E*_2*g*_-like and *A*_1*g*_-like by analogy with MoS_2_ [10]; the *E*_2*g*_-like modes are identified because they have significant in-plane displacements, which would twofold degenerate in hexagonal symmetry, and the modes that have displacements with components predominantly normal to the plane would be non-degenerate and are *A*_1*g*_-like.

We focus on the two modes either side of the gap in the following. In Table 1, we give the frequencies of the Raman-active *A*_*g*_ modes nearest to the gap limits (these are the 8th or 9th and 10th or 11th modes), and the highest frequency Raman-active mode (35th or 36th), and compare to our experimental values. The indices at which *A*_*u*_ (IR) and *A*_*g*_ (Raman) modes occur are not exactly the same for the set of materials or even for the same material with different choices of (for example) pseudopotential, because close-lying *A*_*u*_ and *A*_*g*_ modes can sometimes exchange positions in the sequence. Note that calculations of the phonon dispersion of ReS_2_ show that the gap is not a bandgap, in that it does not extend over the whole Brillouin zone [10], but it is an important and reproducible feature at the zone center. A recent calculation of the phonon dispersion of TcS_2_ used a 2×2×1 supercell, which yields a somewhat better accuracy in the phonon frequencies than the present approach but folds several modes into the range of the gap and so masks its existence in the zone-centre Raman modes [13].

**Table 1:** Calculated zone-centre phonon frequencies of gap-edge and highest Raman-active modes (cm ^−1^)

Mode	TcS_2_	ReS_2_	ReS_2_	ReSe_2_	ReSe_2_
	calc.	calc.	expt.	calc.	expt.
8 or 9	187.9	162.0	161.3	120.9	123.8
10 or 11	252.3	218.2	211.4	152.0	158.2
highest *A*_*g*_	444.27	440.7	438.5	309.6	293.9

**Fig. 2:**
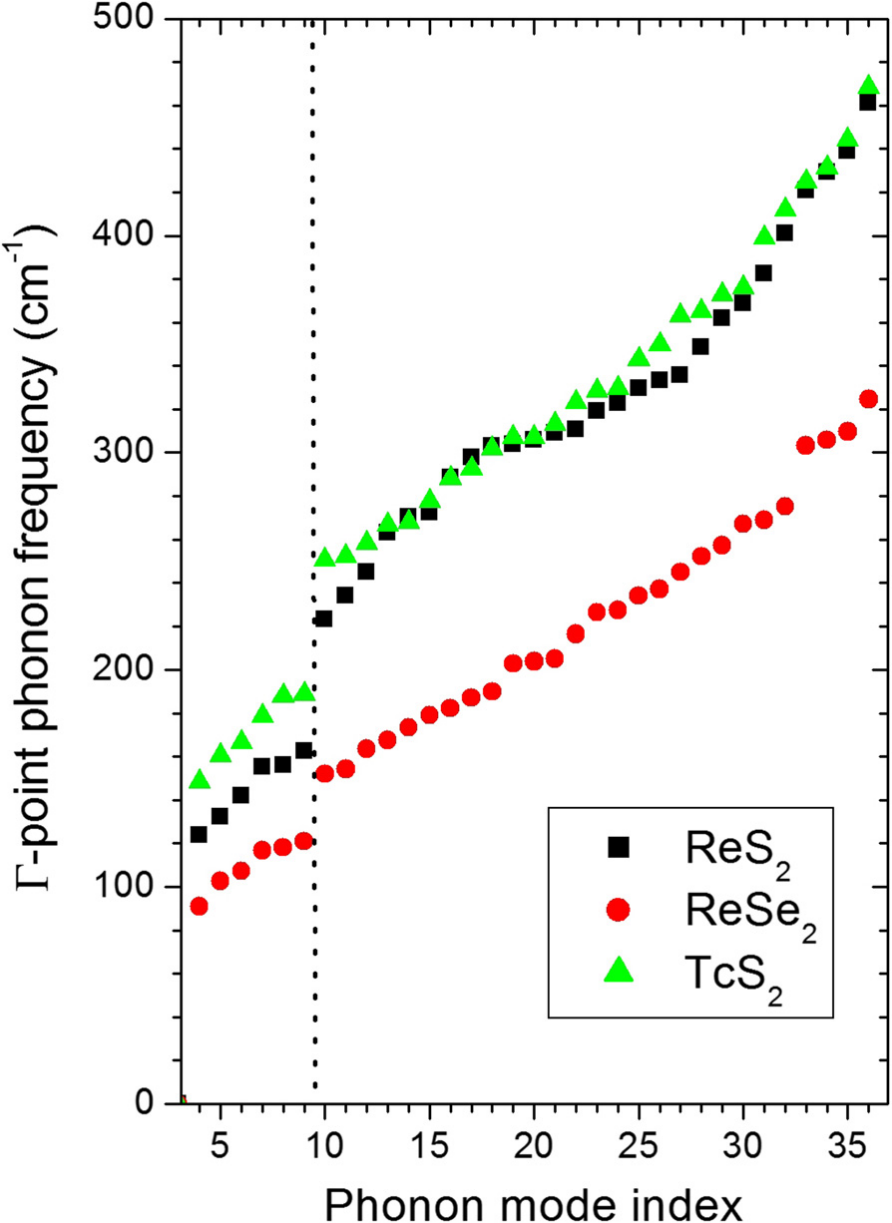
The calculated zone-centre phonon modes of ReS_2_ (*black squares*), ReSe_2_ (*red circles*) and TcS_2_ (*green triangles*) plotted in order of increasing frequency. All modes are either IR- or Raman-active. The *vertical dotted line* indicates the position in frequency of the gap in the phonon spectrum between modes 9 and 10. Note, modes 1–3 are zero-frequency rigid displacements of the unit cell and are not plotted

**Fig. 3:**
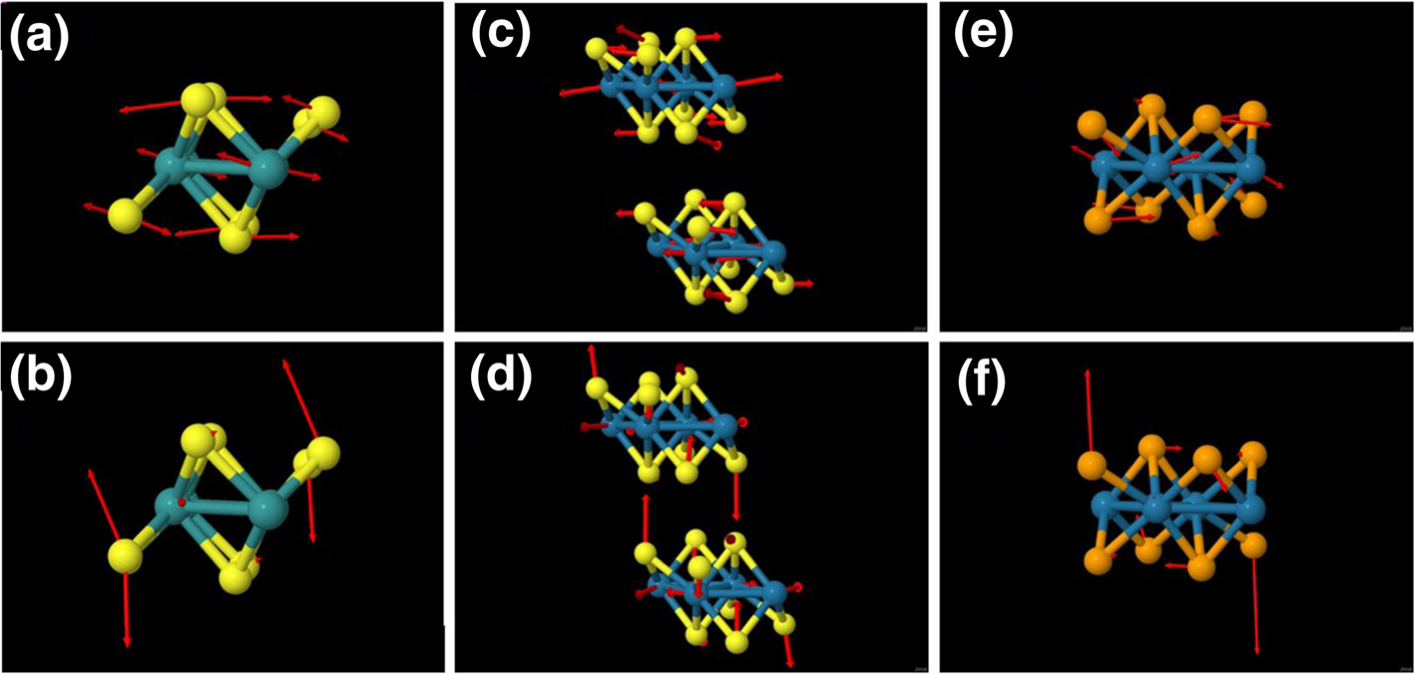
The unit cell and phonon displacement vectors (*red*) for TcS_2_ (**a**, **b**), ReS_2_ (**c**, **d**) and ReSe_2_ (**e**, **f**). *Top row*: displacements for the highest frequency mode (mode 9) below the gap and *bottom row*: displacements for the lowest frequency mode above the gap (mode 10). S, *yellow*; Se, *orange*; Tc, *turquoise*; Re, *blue*

To summarize the DFPT predictions, we plot in Fig. 2 the calculated frequencies of the 33 zone-centre vibrational modes (18 *A*_*g*_ and 15 *A*_*u*_) in order of increasing frequency. The gap occurs in all three materials at the same position, between the 9th and 10th modes, emphasizing the strong similarity between these three compounds. The modes of the sulphides however cover a larger frequency range than those of the selenide, as one would expect based on the lower mass of sulphur (32.065) compared to selenium (78.96), and the frequencies of the Tc and Re sulphides are closely similar well above the gap but different near and below it. This suggests a simple classification of the modes into those in which the displacements of the metal atoms are significant (mainly below the gap) and those where it is predominantly the chalcogens that are moving (mainly above the gap). By considering the predicted atomic displacements for all modes, we can see that this classification is justified, though there are too many modes to show an exhaustive set of displacements here. As an example, we show in Fig. 3 one view of the predicted displacements for the two gap-edge modes (top and bottom rows, respectively). The displacements of the metal atoms are approximately in-plane and are significant for the lower-frequency mode (8 or 9) but smaller for the higher mode; however, for the higher mode (10 or 11), there are significant out-of-plane displacements of the chalcogen atoms. Similar patterns of phonon displacements were presented earlier for ReS_2_ [10] and, as Fig. 3 shows, the displacement patterns are broadly similar for all three compounds. Concentrating on the displacements of the chalcogen atoms, we can also see the in-plane character of mode 9 (*E*_2*g*_-like) and the out-of-plane character of mode 10 (*A*_1*g*_-like), in agreement with earlier work [10], and that this is valid also for TcS_2_.

**Fig. 4:**
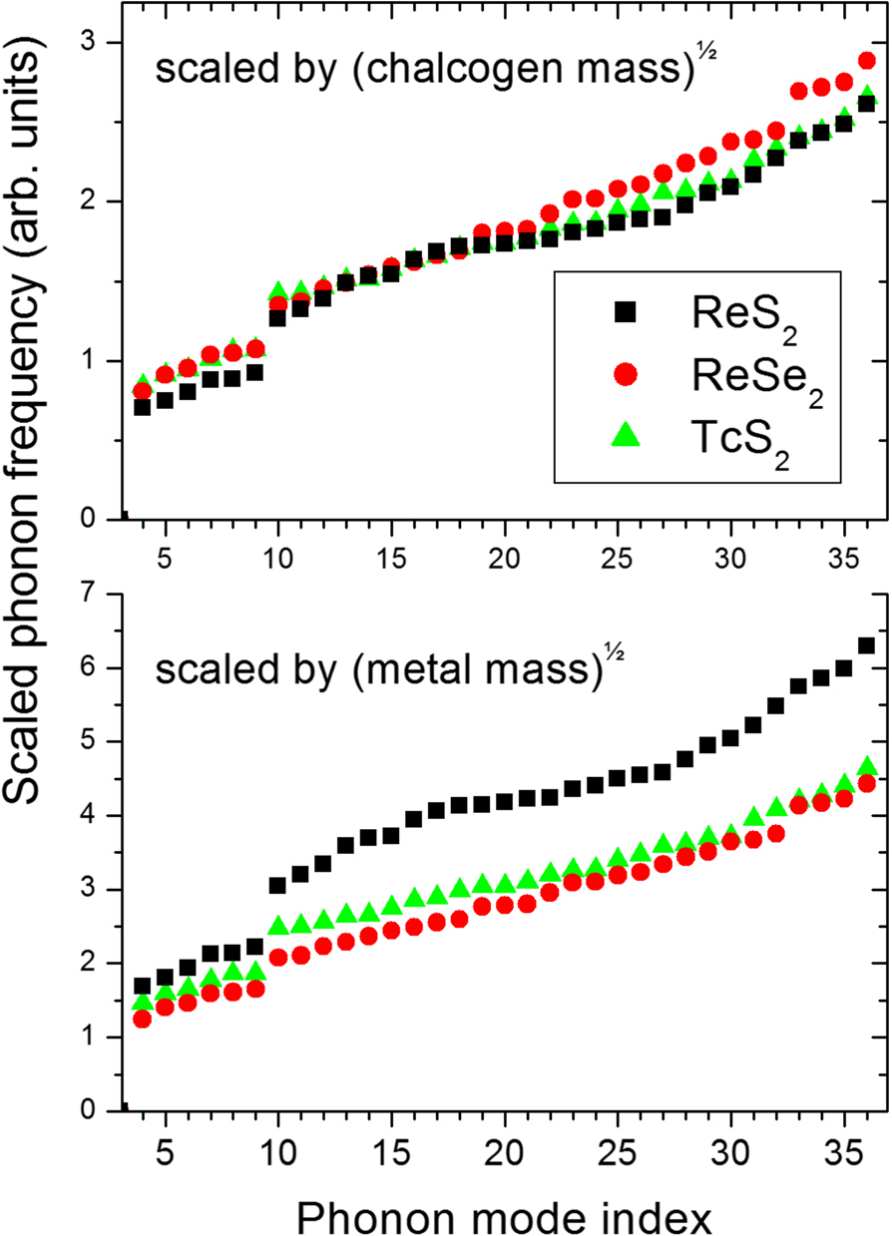
The calculated zone-centre phonon modes of ReS_2_ (*black squares*), ReSe_2_ (*red circles*) and TcS_2_ (*green triangles*) plotted in order of increasing frequency and scaled as discussed. *Top*: scaled by the square root of the chlacogen mass; *bottom*: scaled by the square root of the transition metal mass

The similarity of the calculated displacements suggests that the interpretation above is reasonable. To test this further, we make the very simple assumption that, if interatomic “force constants” *k* are comparable, the frequencies of corresponding displacement patterns will scale with the square root of the mass *m* of the displaced atoms ($\omega \propto \sqrt {k/m}$). For the modes above the gap, this is the chalcogen mass, and therefore, we replot the calculated phonon frequencies in Fig. 4 (top panel) multiplied by $\sqrt {32.065}=5.66$ (sulphides) and by $\sqrt {78.96}=8.89$ (selenide). The effect of this scaling is dramatic; all three sets of phonon modes now almost coincide above the gap. We can also test the idea that the modes below the gap should scale with the square root of the reduced mass taking into account the metal ions (Fig. 4, bottom panel); the displacement patterns of Fig. 3 suggest that this will not be so successful, because both metal and chalcogen atoms are displaced significantly in the below gap modes. However, by comparison of Fig. 4 with Fig. 2, it is clear that the ReS_2_ and TcS_2_ modes 4–9 now lie closer together.

Finally, in Table 2, the calculated zone-centre phonon frequencies of TcS_2_ are given, since these have not been presented elsewhere. We also give the predicted infrared and Raman activities of these modes, principally to identify the Raman-active modes of the set; it should be noted that the Raman activity given here is the one computed in Quantum Espresso and other packages and represents a specific experimental geometry and an average over all possible sample orientations, as given in Eq. (6) of Porezag et al. [29]. So it does not correspond to the experimental configuration for a cleaved flake supported on a substrate; however, it would be relevant to the measurements on a polycrystalline or colloidal sample, the forms in which TcS_2_ is likely to be available.

**Table 2:** Calculated zone-centre phonon frequencies of TcS_2_

Index	Frequency (cm ^−1^)	IR Activity	Raman Activity
4	148.14	0.0000	428
5	160.38	0.0000	1016
6	166.40	0.0484	0
7	178.54	0.0000	3964
8	187.93	0.0000	900
9	188.51	0.1416	0
10	250.77	0.3533	0
11	252.28	0.0000	3967
12	258.18	0.3939	0
13	266.53	0.0000	505
14	267.82	1.2843	0
15	277.76	0.0000	806
16	287.93	0.3621	0
17	292.34	0.0000	4655
18	301.69	0.0000	2999
19	306.90	0.1785	0
20	307.26	0.0000	3179
21	312.84	0.0000	2761
22	323.05	1.6289	0
23	328.50	0.0000	1318
24	329.73	4.2948	0
25	343.13	2.9997	0
26	349.84	0.0000	4650
27	362.87	0.1476	0
28	365.12	0.0000	961
29	372.55	0.2031	0
30	375.97	0.0000	5592
31	399.18	0.3195	0
32	411.93	0.0000	6409
33	424.81	0.0000	19,660
35	444.27	0.0000	9908
36	468.57	3.9835	0

## Conclusions

We have shown that a simple argument based on scaling by chalcogen mass allows one to understand the zone centre phonon modes of the rhenium and technetium dichalcogenides in a unified way and to understand qualitatively the distribution of the modes in frequency. This emphasizes the strong similarity between the members of this family of materials. The good agreement between the predicted and experimental frequencies of the Raman bands of the rhenium dichalcogenides gives us confidence in the behavior predicted for the technetium dichalcogenides, and this may be of use in monitoring the chemical composition of technetium-containing suspensions and colloids.
